# Trigging stepwise-strand displacement amplification lights up numerous G-quadruplex for colorimetric signaling of serum microRNAs

**DOI:** 10.1016/j.isci.2023.106331

**Published:** 2023-03-04

**Authors:** Huo Xu, Fenglin Yang, Danlong Chen, Weilin Ye, Guohui Xue, Lee Jia

**Affiliations:** 1 College of Materials and Chemical Engineering, Minjiang University, Fuzhou, Fujian, 350108, China; 2 Department of Clinical Laboratory, Jiujiang NO.1 People’s Hospital, Jiujiang, Jiangxi, 332000, China

**Keywords:** Analytical chemistry applications, Diagnostic technique in health technology, Medical device in health technology

## Abstract

MicroRNAs (miRNAs) play an important biomarker in various biological processes, especially cancer related, yet economic, simple, sensitive and specific methods for miRNA determination are still challenging. In this study, we have developed stepwise-strand displacement amplification (S-SDA)-based colorimetric sensing platform for let-7a miRNA detection in clinical serum samples. Our results demonstrated that the developed S-SDA-based method shows high sensitivity with a detection limit of 63.2 pM and a naked eye detection limit of 0.1 nM. Moreover, the S-SDA amplifier is able to discriminate target miRNAs from their mutants with high accuracy and specificity. With its high sensitivity and selectivity, this method successfully identified healthy individuals from patients with colon cancer by detecting let-7a miRNAs in serum. We believe the colorimetric analysis method will provide a new paradigm for the detection of miRNA with different abundance and show great potential for clinical application in biomedical analysis and early clinical diagnosis.

## Highlights

• A highly specific and sensitive machinery for colorimetric miRNA detection is proposed
• The LOD for naked eye detection was estimated to be 0.1 nM
• Our method can be further used to detect let-7a in clinical serum samples
• S-SDA has significant potential in biochemical studies and clinical diagnosis

## Graphical abstract

**Figure:**
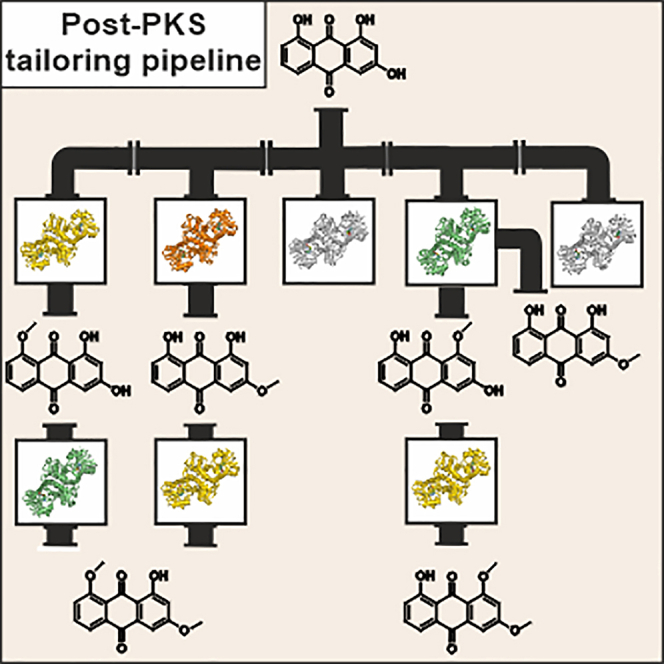

## Introduction

MicroRNAs (miRNAs) are a class of endogenous non-coding RNAs (ca. 18–24 nt), and as important gene regulators, they are widely involved in a series of biological processes, such as cell differentiation, expression, cellular proliferation, and so on.^1^^,^^2^ A large number of studies have shown that the abnormal expression of miRNA is linked to the pathogenesis of a variety of human genetic diseases,^3^^,^^4^^,^^5^^,^^6^ such as cancer, cardiovascular disease, diabetes, and neurodegenerative diseases.^7^^,^^8^^,^^9^ Therefore, quantitative detection and analysis of miRNAs expression is of great significance for early diagnosis and treatment of related diseases. However, due to the short length, low abundance, and high sequence similarity of family members, it makes difficulties to quantify the expression level of miRNAs.

So far, researchers have disclosed different detection techniques and methods, including PCR,^10^ northern blotting,^11^ and DNA microarrays.^12^ However, these traditional nonamplified methods always need high criteria for instruments, high cost, complicated operation, poor sensitivity, and false-positive results. Therefore, new signal amplification strategies method for analyzing miRNAs has been developed, including hybridization chain reactions,^13^ catalytic hairpin assembly,^14^ strand displacement amplification (SDA),^15^^,^^16^ and rolling circle amplification.^17^^,^^18^ Especially, SDA has become an attractive tool for quantitative detection and analysis of miRNAs expression in the past decade due to its simple operation, isothermal reaction, rapid amplification kinetics, and high sensitivity. However, most of the reported SDA-based methods usually require the labeling of fluorescence, organic dyes, quantum dots, or nanoparticles in these designs, which leads to the increase of the cost and sophisticated processes. In an alternative approach, the visual sensing miRNA has become a compelling emerging research field in recent years due to its easy handling and commercialization. Recently, the G-quadruplex-hemin DNAzyme that is similar to horseradish peroxidase could catalyze the oxidation of 3,3′,5,5′-tetrazmethylbenzidine sulfate (TMB) or 2,2′-azinobis(3-ethylbenzthiazoline-6-sulfonic acid disodium salt) (ABTS^2−^) by H_2_O_2_^19^ has been increasingly used for colorimetric,^20^ electrochemical,^21^ and/or chemiluminescence^22^ detection of a variety of target, including DNA, protein, metal ion, and telomerase activity. As compared with protein enzymes, the intrinsic merit of DNAzymes depends on their versatility of design and low cost. However, the SDA-based method always suffers from low signal-to-noise ratio problems due to inevitable background amplification,^23^ which limits the realization of detection sensitivity.

Here, we wish to develop a colorimetric-based autonomous polymerization/nicking machinery for miRNA (using let-7a miRNA as a model) detection. By coupling G-quadruplex DNAzyme-based sensing systems with the significant amplification efficiency of SDA, let-7a miRNA could trigger the stepwise-SDA (S-SDA) to form plenty of G-quadruplex, providing an amplified readout signal of the target miRNA and, thus, offering high detection sensitivity. Meanwhile, due to the excellent discriminative ability of S-SDA employed, the high specificity of this method was obtained. In addition, this strategy has been successfully developed for colorimetric analysis of let-7a miRNA in a complex condition and clinical serum samples. With these advantages, this efficient S-SDA-based autonomous machinery holds a significant potential in biochemical studies and clinical diagnosis.

## Results

### Principle of the proposed strategy

**Scheme 1:**
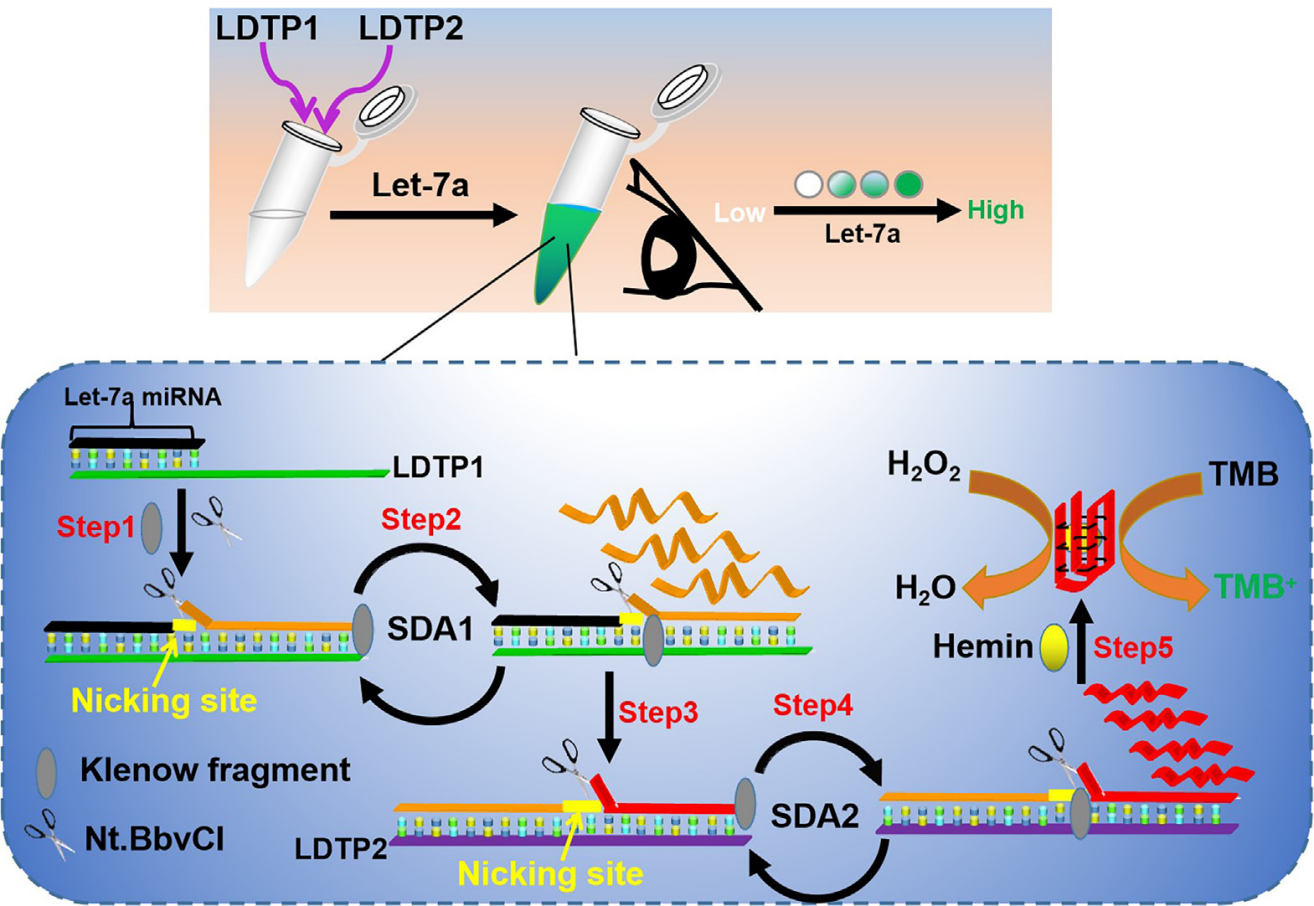
Schematic illustration of the stepwise-strand displacement amplification (S-SDA) mediated by klenow fragment polymerase (KF) for colorimetric detection of target let-7a miRNA

The principle of target let-7a miRNA-induced S-SDA is illustrated in Scheme 1. Briefly, this system mainly consists of only two linear DNA template probe (LDTP1 and LDTP2), DNA polymerase, and nicking endonuclease. The LDTP1 consists of two domains: ① let-7a miRNA recognition region and ② a recognition domain for Nt.BbVCI, which could recognize (5′-CCTCAGC-3′/3′-GGAGTCG-5′) and specifically cleave the upper double-stranded DNA (dsDNA). In the presence of target miRNA, it will specifically bind to LDTP1, and the 3′ end of miRNA can be regarded as a polymerized primer to initiate polymerization with the help of KF polymerase and dNTPs, which synthesizes a duplex conformation. Then, nicking endonuclease Nt.BbVCI specifically cleaves the upper DNA duplex. Through the repeated extension and cleavage via SDA1 reaction, abundant DNA triggers are displaced. Furthermore, the released DNA triggers are free to bind to LDTP2 to initiate the second extension and cleavage cycle via SDA2 reaction. This S-SDA operation would allow for the production of plenty of DNAzymes that triggers the subsequent oxidation of TMB by H_2_O_2_ in the presence of hemin for colorimetric signal transduction. Conversely, the S-SDA reaction cannot be initiated and the hemin/G-quadruplex DNAzyme structures cannot be formed without target miRNA. Due to the high amplification efficiency of SDA1 and SDA2, one target miRNA could be amplified for colorimetric detection with high sensitivity.

### Feasibility study

**Figure 1:**
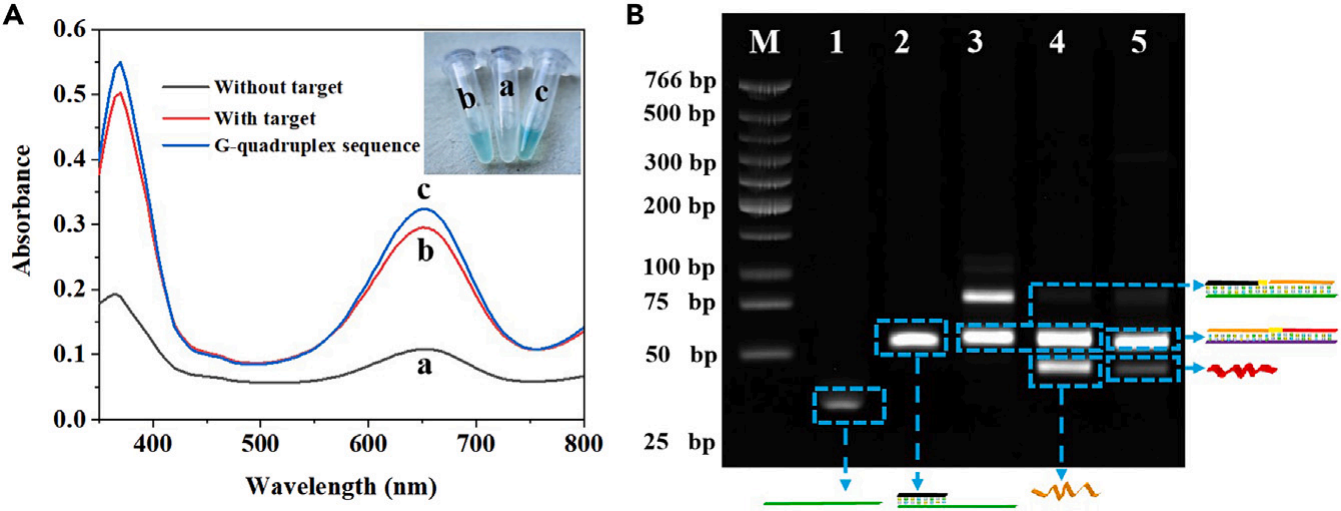
The feasibility of S-SDA-based visible detection for let-7a miRNA (A) The absorbance spectra of the label-free colorimetric sensing platform for miRNA detection under different condition. (a: without miRNA; b: with miRNA; c: G-quadruplex sequence; inset: the corresponding photographs of the solution color change). (B) 12% native-PAGE characterization. Lane 1, LDTP1; lane 2, LDTP1 + let-7a miRNA; lane 3, LDTP1 + let-7a miRNA+KF; lane 4, LDTP1 + let-7a miRNA+KF+Nt.BbvCI; lane 5, LDTP1 + let-7a miRNA+KF+Nt.BbvCI+LDTP2.

To verify the feasibility of the proposed method for let-7a miRNA analysis, UV-vis spectra were used. As shown in Figure 1A, in the case of no target miRNA, the G-quadruplex sequence was almost difficult to form and the absorption peak (650 nm) was very low (curve a), indicating that no S-SDA reaction executes and no DNAZyme is produced. Whereas obvious enhanced absorption was observed in the presence of let-7a miRNA (curve b), which is slightly lower than with the use of G-quadruplex sequence only (curve c). These results indicated the occurrence of S-SDA reactions and generation of some hemin/G-quadruplex DNAzyme upon the addition of target miRNA. This UV-vis spectra result was in good agreement with the result of naked eye observation. As shown in Figure 1A **inset**, a clear green color was observed by the naked eye in the presence of let-7a miRNA, indicating the detection and discrimination process can be seen with the naked eye without the need of any complicated measuring instrument. The designed S-SDA fabrication and recognition process has also been confirmed by 12% non-denaturing PAGE. As shown in Figure 1B, the bands of LDTP1 are shown in lane 1. The bright band in lane 2 could be attributed to the hybridization of the LDTP1 and let-7a miRNA (LDTP1/let-7a miRNA). With addition of the KF and dNTPs, a dsDNA with the recognition site for Nt.BbvCI that moved slower than LDTP1 was observed (lane 3), indicating that let-7a miRNA can hybridize with the LDTP1 to initiate the extension from the 3′ end to produce the dsDNA intermediates. When the reaction is conducted in the presence of Nt.BbvCI and KF, the dual bands (lane 4), consisting of a band matching the above dsDNA and another band matching the DNA trigger1, show the SDA1 reaction occurs with the assistance of KF and Nt.BbvCI. Lane 5 shows the electrophoresis result of addition of all compounds (LDTP1, LDTP2, KF, and Nt.BbvCI). As expected, the SDA2 reaction is triggered to form a dsDNA (LDTP2/DNA trigger1 extension, first band in lane 5) and the DNA trigger2 (G-quadruplex mixture, last band in lane 5). These results shown here clearly demonstrate that the proposed colorimetric method can be used for miRNA detection via simple way.

### Sensitivity of miRNA assay

**Figure 2:**
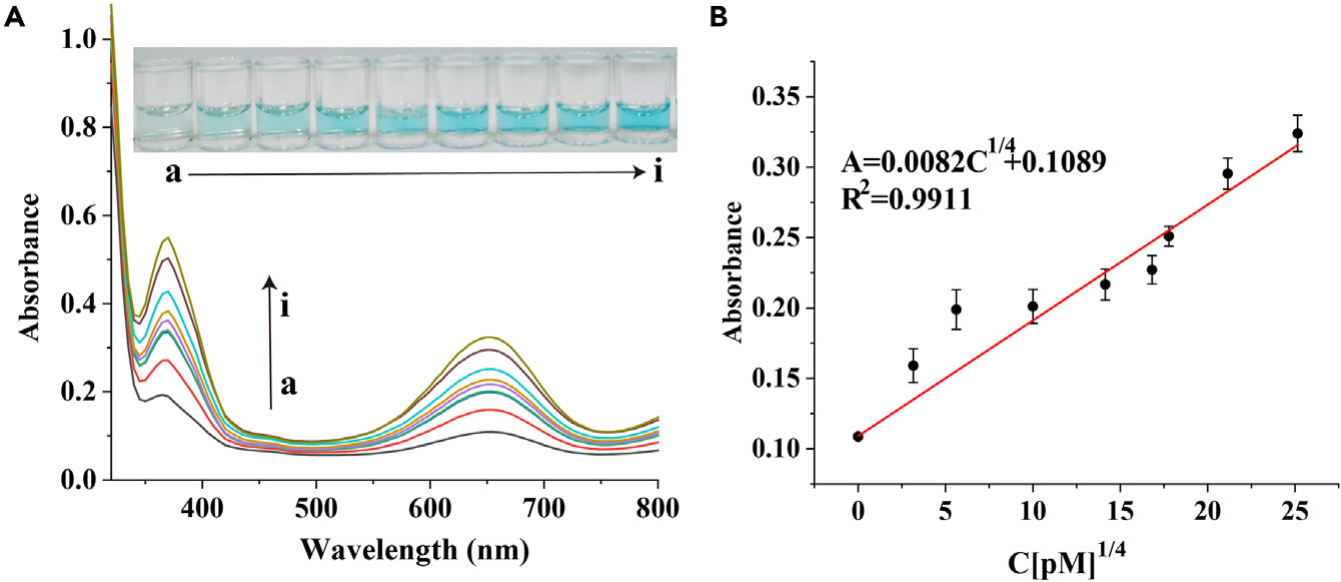
Sensitivity of miRNA detection (A) The photographs (inset) and UV-vis absorption spectra of colorimetric sensor analyzing different concentrations of let-7a miRNA (from a to i): 0, 0.1, 1, 10, 40, 80, 100, 200, 400 nM. (B) Relationship between the absorption at 650 nm and the concentration of let-7a miRNA. Correlation of absorption at 650 nm and [let-7a miRNA] ^1/4^. Error bars represent the standard errors (n = 3).

Under the optimized experimental conditions (Figure S1), a series of different concentrations of target miRNA (0–400 nM) were measured to estimate the sensitivity and linear range of the colorimetric method for let-7a miRNA detection. As shown in Figure 2A, it can be observed clearly that the UV-vis absorption intensity increases with the increase of let-7a miRNA concentration from 0 to 400 nM and the solution color changes from colorless to green with the increasing amount of added let-7a miRNA. Notably, in the range from 0 to 400 nM, the absorption peak value at 650 nm shows a linear correlation with 1/4th root of concentration of let-7a miRNA (Figure 2B). The correlation equation is A = 0.0082C^1/4^ + 0.1089 (correlation coefficient was 0.9911), where A and C are the absorption peak value at 650 nm and the concentration of let-7a miRNA (pM), respectively. According to the equation the limit of detection (LOD) = the average absorption value of the blank + three times the standard deviation, LOD was calculated to be 63.2 pM for instrument detection. Such sensitivity is superior or comparable to many other enzyme-assisted amplified sensors (Table S2). Notably, there occurred distinct color change upon addition of 0.1 nM. Therefore, the LOD for naked eye detection was estimated to be 0.1 nM. This is attributed to the high efficiency of S-SDA for signal amplification, making it more promising for application in early diagnosis with high sensitivity.

### Detection specificity

**Figure 3:**
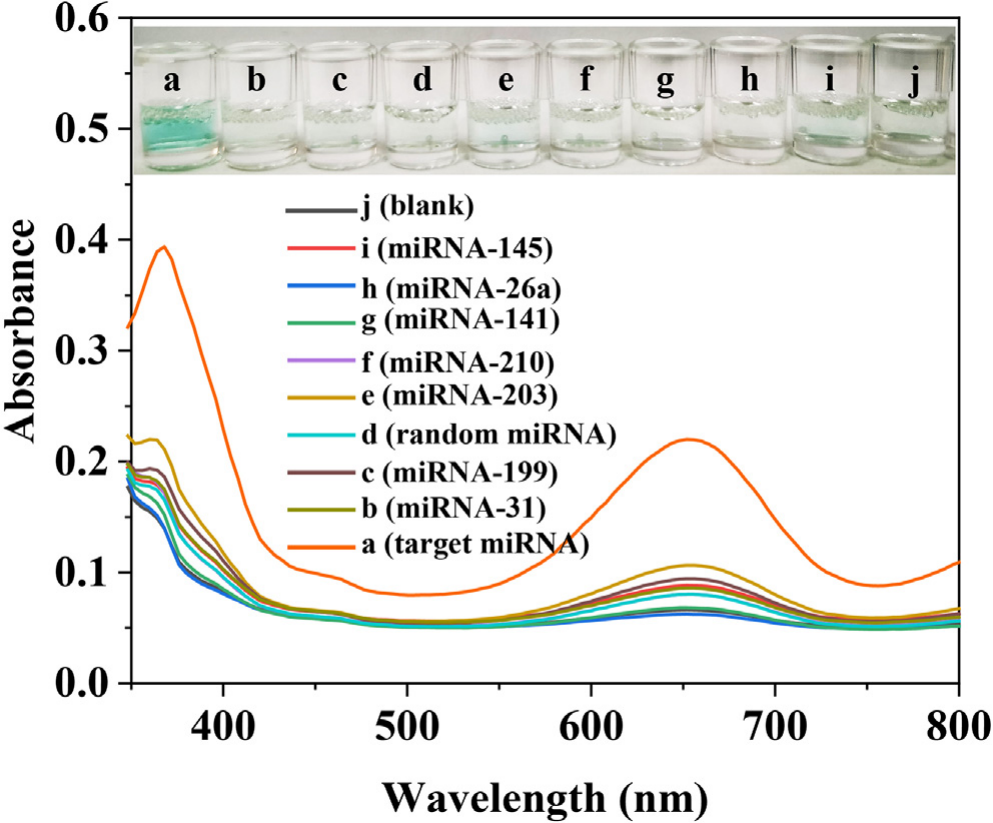
UV-vis spectra of samples showing the discrimination of let-7a miRNA against other miRNAs Inset: the corresponding the color change of the samples.

To assess the selectivity of the developed S-SDA, different miRNAs, including let-7a, miRNA-31, miRNA-199, miRNA-203, miRNA-210, miRNA-141, miRNA-26a, miRNA-145, and random miRNA, are selected as the detection model. As shown in Figure 3, noncomplementary targets as interfering agents (miRNA-31, miRNA-199, miRNA-203, miRNA-210, miRNA-141, miRNA-26a, miRNA-145, and random miRNA) do not cause absorbance signal changes, which was similar to that of the blank, but significant absorbance intensity at 650 nm was observed in the presence of target miRNA (let-7a miRNA). Similarly, the color of the solution shows the same trend. As shown in Figure 3 **inset**, a remarkably distinguishable color change can also be observed for let-7a miRNA compared with other miRNAs. To further investigate the specificity of the S-SDA-based method, the nonspecific miRNAs of the other members of let-7 family (let-7b, let-7c, let-7d, and let-7i) were tested with the same concentration. As shown in the Figure S2, compared with the let-7a miRNA, the absorbance signal or color changes of the miRNA family members with one, two, or four mismatched nucleotides showed nearly negligible. These results above indicated that our S-SDA-based colorimetric sensing platform has superb selectivity for let-7a miRNA toward other competitive miRNAs.

### Detection of let-7a miRNA in real samples

**Figure 4:**
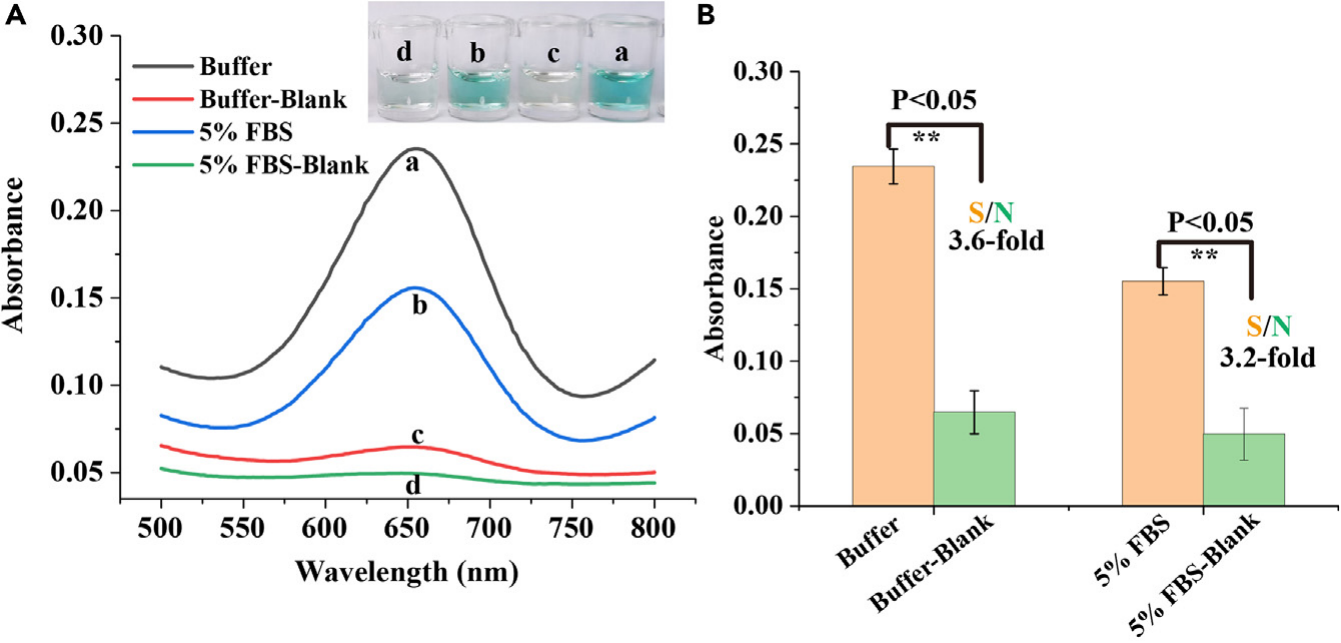
Investigation of matrix effect on the stability of S-SDA-based sensing platform for let-7a miRNA detection (A) Comparing absorption spectra of the proposed strategy for let-7a detection in buffer and 5% fetal bovine serum. Inset: the corresponding color change of the samples. (B) The absorbance at 650 nm as derived from (A). ∗∗ = p < 0.05. Data represented as mean ± SD.

To investigate the practicality of the S-SDA-based colorimetric sensing platform for miRNA detection in real biological samples, let-7a miRNA was added into the commercial fetal bovine serum (FBS) and absorbance measurements were monitored. As shown in Figure 4, there was no statistically significant change in the S/N (approximately 3.4) after their incubation in 0% and 5% FBS. To further test the practical utility of S-SDA-based sensing platform, various concentrations of let-7a miRNA were added in 1000-fold and 100-dilution of human serum samples that obtained from healthy person. The detected results were shown in Table S3 and exhibited good recovery. These results indicate that the developed S-SDA-based colorimetric sensing platform can be successfully applied to complex biological environment.

**Figure 5:**
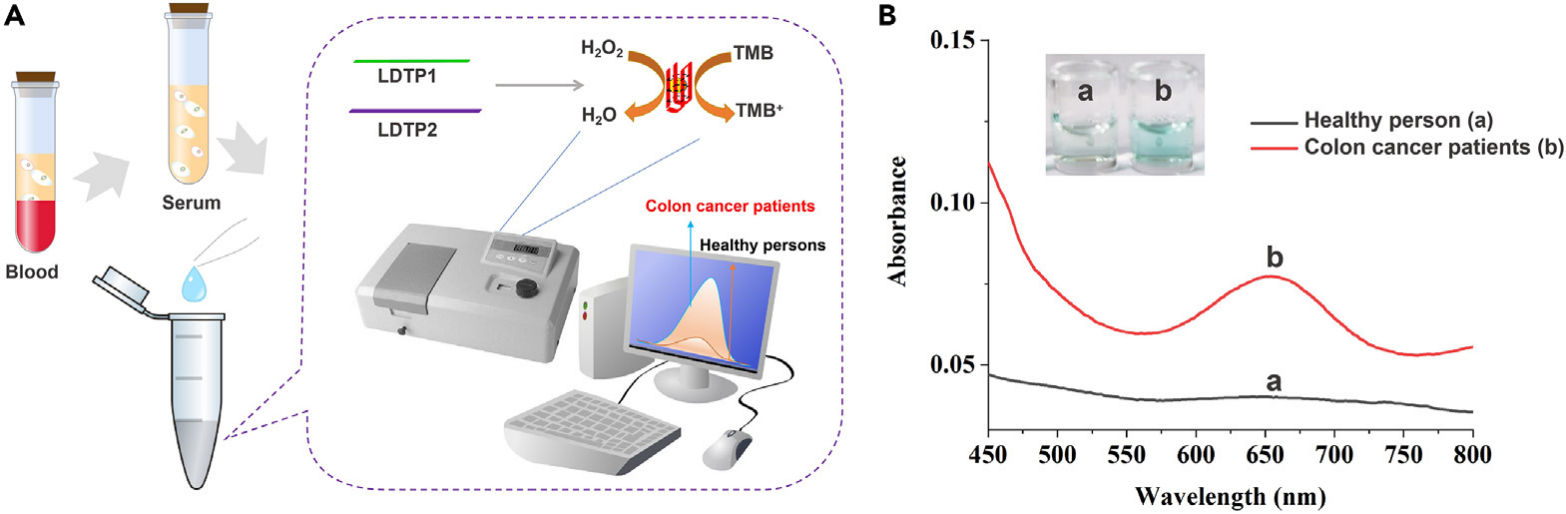
Let-7a miRNA validation and application on clinical specimens (A) Schematic illustration of the principle of miRNAs detection in human serum by S-SDA. (B) Results of the absorbance spectra and photograph (inset) of sensing platform for colon cancer-related let-7a miRNA detection in clinical serum samples.

To further demonstrate the practical application of the S-SDA-based colorimetric sensing platform, serum samples derived from patients with colon cancer and non-cancerous healthy serum sample (healthy person) which obtained from Jiujiang First People’s Hospital, and absorbance intensity was monitored (Figure 5A). In order to simplify the detection, blood samples were centrifuged to obtain the corresponding serum, and then heated at 95°C for 3 min to inactivate interfering nucleic acids-degrading enzymes that might interfere with S-SDA detection. As shown in Figure 5B, the absorbance of patients’ sample with colon cancer was significantly higher compared to non-cancerous healthy sample. The obtained data indicated that let-7a miRNAs are overexpressed in cancer samples. What is pleasant is that patients with cancer are easily distinguished from healthy people with the naked eye by using the method (Figure 5B **inset**). Furthermore, miRNAs were detected blindly in order to demonstrate S-SDA’s ability to distinguish target miRNA from nontarget miRNA. Based on their relative absorption intensities, target miRNA (let-7a miRNA) and nontarget miRNA can be distinguished from one another in Table S4. Assay results from the blind test were in agreement with laboratory assistant’s pre-made marks. These preliminary results demonstrated that the S-SDA-based colorimetric sensing platform could be useful in clinical settings for colon cancer diagnosis by using clinical serum samples.

## Discussion

In summary, we have developed S-SDA-based colorimetric sensing platform for let-7a miRNA detection. Taking advantage of the high amplification efficiency of dual-stage isothermal amplification reaction, highly sensitive visual detection of let-7a miRNA by the naked eye without using any sophisticated instruments was achieved. We have proved that S-SDA-based method exhibited excellent selectivity and was successfully applied to detect let-7a miRNA in a complex matrix samples including the FBS and clinical serum samples. On the basis of the above analytical characteristics, we believe the naked eye detection based on S-SDA colorimetric sensing platform is expected to have potential applications in biomedical detection and clinical diagnostics.

### Limitation of the study

In this study, let-7a miRNA levels were found to be higher in patients with colon cancer than in healthy people; however, the accuracy of this approach needs to be verified in case of the real applications due to the interference of other substances in blood.

## STAR★Methods

### Key resources table

**Table:** 

REAGENT or RESOURCE	SOURCE	IDENTIFIER
**Chemicals, peptides, and recombinant proteins**		

Klenow fragment exo^-^ DNA polymerase	New England Biolabs Inc.	M0212L
Nt. BbvCI nicking endonuclease	New England Biolabs Inc.	R0632L
H_2_O_2_	Aladdin	H112515
TMB	Aladdin	T274310

**Deposited data**		

Bulk RNAseq		NCBI’s Gene Expression Omnibus GSE179220
Let-7a miRNAseq	This paper	NCBI’s Gene Expression Omnibus GSE387244

**Experimental models:** Organisms/Strains		

Colon cancer patients	Jiujiang First People's Hospital	JJSDYRMYY-YXLL-2021-016
Healthy person	Jiujiang First People's Hospital	JJSDYRMYY-YXLL-2021-016

**Software and algorithms**		

DNA analysis	NUPACK	https://www.nupack.org/
Origin	OriginLab Corporation	https://www.originlab.com/
Prism 7	Graph Pad	https://www.graphpad.com/

### Resource availability

#### Lead contact

Further information and requests for resources and materials should be directed to and will be fulfilled by the lead contact, Prof. Huo Xu (chemicalxuhuo@163.com).

#### Materials availability

This study did not generate new unique reagents.

### Experimental model and subject details

A total of 10 plasma samples from colon cancer patients (median age: 61 years old, range: 48-72; Male: Female = 7:3), and 10 plasma samples from healthy person (median age: 60 years old, range: 50-71; Male: Female = 7:3) were collected in November 2022 from Jiujiang First People's Hospital, China. This study was approved by the Ethics Committee of Jiujiang First People's Hospital (JJSDYRMYY-YXLL-2021-016). All participants signed a consent form for participation in the survey, with a permission for sample collection, utilization, and data analysis.

### Method details

#### Materials

The oligonucleotides used in this work were synthesized by Sangon Biotech Co., Ltd. (Shanghai, China) and their sequences are shown in Table S1, which were diluted in 1×TE buffer (10 mM Tris-HCl, 1 mM EDTA, 12.5 mM MgCl_2_, pH 8.0) to obtain the 10 μM stock solution. The Klenow fragment exo^-^ DNA polymerase and Nt. BbvCI nicking endonuclease were obtained from New England Biolabs Inc. (USA). Total RNAs of MCF-7 cells was extracted with cell total RNA isolation kit V2 (RC112) (Vazyme, Nanjing).

#### Instrumentation

The UV-vis absorbance measurements were performed on a Specord 210 plus UV-vis spectrophotometer (Jena, Germany) at room temperature. Photos were taken using a Huawei P30 pro.

#### Gel electrophoresis

The electrophoresis was carried out on 12% nondenaturing polyacrylamide gels to verify the products of S-SDA. Electrophoresis was performed in 0.5× Tris-borate-EDTA (TBE) (pH 8.0) at a constant voltage of 80 V for 1 h at room temperature. The resulting gel was photographed by gel image system.

#### miRNA assay

The target miRNA determination could be briefly described as follows: 0.5 μL of 10 μM linear DNA template probe 1 (LDTP1) was first added to the mixture of 1 μL of different concentrations of target miRNA and 20 μL of 1×NEB buffer2, followed by incubating at 90°C for 3 min and then allowed to cool to room temperature for 1 h before use. Then, S-SDA reaction occurred by adding 0.5 μL of 10 μM linear DNA template probe 2 (LDTP2), 0.5 μL of 5 U/μL Klenow Fragment exo^-^, 0.5 μL of 10 U/μL Nt.BbvCI, and 1 μL of 10 mM dNTPs and then were incubated at 37°C for 2 h. Subsequently, the reaction was stopped by keeping the resulting solution at 80°C for 20 min.

Subsequently, 2 μL of 50 μM hemin and 86.5 μL 2×HEPES buffer were added into 25 μL of the above-mentioned reaction and incubated for 1 h to facilitate the formation of hemin/G-quadruplex HRP-mimicking DNAzyme. After that, 5 μL of 25 mM TMB and 2.5 μL 20 mM H_2_O_2_ were rapidly injected into the resulting solution to initiate the colorimetric reaction and and incubated at room temperature for 10 min. Finally, the absorbance of the solution was measured by UV-vis characterization and a visual photograph of the solution was taken using a Huawei P30 pro.

### Quantification and statistical analysis

GraphPad Prism7.0 was used to all statistical analysis and data visualization. Origin software (2019b) was used to compile and analyze data.Significance levels were defined as following: ns, P > 0.05, ∗∗P < 0.05. Data are represented as mean ± SD.

### Additional resources

The clinical experiment was approved by the Ethics Committee of Jiujiang First People's Hospital (JJSDYRMYY-YXLL-2021-016).

## Data Availability

• In this study, small RNAseq data generated have been deposited on NCBI's GEO Datasets platform (accession number: GSE179220) and are publicly available as of the date of publication. The let-7a miRNAseq data have been published previously (NCBI GEO; accession number: GSE387244). • This paper does not report original code or reagent. All codes or reagents used in this study are listed in the key resources table. • Any additional information required to reanalyze the data reported in this paper is available from the lead contact upon request.
