# Supplementary material for: Trigging stepwise-strand displacement amplification lights up numerous G-quadruplex for colorimetric signaling of serum microRNAs
Source: iScience. 2023 Mar 4;26(4):106331. doi: 10.1016/j.isci.2023.106331 (PMC10034434; doi:10.1016/j.isci.2023.106331)
Supplement: Document S1. Tables S1–S4 and Figures S1 and S2 [file mmc1.pdf]

iScience, Volume *26*

Supplemental information

Trigging stepwise-strand displacement amplification lights up numerous G-quadruplex for colorimetric signaling of serum microRNAs

Huo Xu, Fenglin Yang, Danlong Chen, Weilin Ye, Guohui Xue, and Lee Jia

Supplemental Information

Table S1. DNA sequences designed in this work[a]. Related to STAR Methods.

Name	Sequence (5'-3')
Linear DNA template probe 1 (LDTP1)	CCCTATAGTGAGTCGTATTAATC**GCTGAGG**CA ACTATACAACCTACTACCTCA
Linear DNA template probe 2 (LDTP2)	CCCAACCCGCCCTACCC**GCTGAGG**TCCCTAT AGTGAGTCGTATTAATC
Let-7a miRNA	UGAGGUAGUAGGUUGUAUAGUU
MiRNA-31	AGGCA AGAUG CUGGC AUAGCU
MiRNA-210	CUGUGCGUGUGACAGCGGCUGA
MiRNA-26a	UUCAAGUAAUCCAGGAUAGGCU
MiRNA-141	CAUCUUCCAGUACAGUGUUGGA
MiRNA-199	ACAGUAGUCUGCACAUUGGUUA
MiRNA-203	GUGAAAUGUUUAGGACCACUAG
MiRNA-145	GUCCAGUUUUCCCAGGAAUCCCU
Random miRNA	GCAGUUGACCAUAUAGCCUGGAUCCA

[a]For LDTPS, the bold letters represent the recognition site of Nt.BbVCI.

Table S2. Comparison with relative works for miRNA detection. Related to Figure 2.

Detection method	Materials	Reaction time	Detection limit	Reference
Fluorescence spectroscopy	MB-GO	1.5 h	400 pM	1
Fluorescence spectroscopy	DNA nanomachine	1 h	80 pM	2
Fluorescence spectroscopy	DNAzyme nanotweezer	2 h	1.5 nM	3
Fluorescence spectroscopy	Split-DNAzyme/AuNP	1.4 h	10 pM	4
Fluorescence spectroscopy	Gold nanoflares	1 h	300 pM	5
Colorimetric assay	Enzyme-free signal amplification in the DNAzyme sensor via target-catalyzed hairpin assembly	~3 h	20 pM	6
Colorimetric assay	Palindromic molecule beacon-based cascade amplification	~3 h	10 pM	7
Electrochemical method	Ligase chain reaction amplification	~100 h	12 fM	8
Colorimetric assay	S-SDA based nanomachine	3.5 h[a]	63.2 pM	Our approach

[a]Although the slightly longer assay time was needed, no other materials (e.g., electrode, gold nanoparticles and graphene oxide) are involved and improved assay capability is achieved. Additionally, the assay experiments are performed in homogenous solution and only several mixing steps are involved.

1. Yang, L., Liu, B., Wang, M., Li, J., Pan, W., Gao, X., Li, N., Tang, B. (2018). A Highly Sensitive Strategy for Fluorescence Imaging of MicroRNA in Living Cells and in Vivo Based on Graphene Oxide-Enhanced Signal Molecules Quenching of Molecular Beacon. ACS Appl. Mater. Interfaces 10, 6982-6990.
2. Duan, L., Liu, J., Yu, R., Jiang, J. (2021). DNAzyme Cascade Circuits in

Highly Integrated DNA Nanomachines for Sensitive microRNAs Imaging in Living Cells. Biosens. Bioelectron. 177, 112976.
3. He, M., He, M., Nie, C., Yi, J., Zhang, J., Chen, T., Chu, X. (2021). mRNA-Activated Multifunctional DNAzyme Nanotweezer for Intracellular mRNA Sensing and Gene Therapy. ACS Appl. Mater. Interfaces. 13, 8015-8025.
4. Wu, Y., Huang, J., Yang, X., Yang, Y., Quan, K., Xie, N., Li, J., Ma, C., Wang, K. (2017). Gold Nanoparticle Loaded Split-DNAzyme Probe for Amplified miRNA Detection in Living Cells. Anal. Chem. 89, 8377-8383.
5. Liu, L., Li, N., Huang, Z., Tang, L., Ying, Z., Jiang, J. (2020). Gold Nanoflflares with Computing Function as Smart Diagnostic Automata for Multi-miRNA Patterns in Living Cells. Anal. Chem. 92, 10925-10929.
6. Zheng, A., Li, J., Wang, J., Song, X., Chen, G., Yang, H. (2012). Enzyme-free signal amplification in the DNAzyme sensor via target-catalyzed hairpin assembly. Chem. Commun. 48, 3112-3114.
7. Shen, Z., Li, F., Jiang, Y., Chen, C., Xu, H., Li, C. (2018). Palindromic molecule beacon-based cascade amplification for colorimetric detection of cancer genes. Anal. Chem. 90, 3335-3340.
8. Zhu, W., Su, X., Gao, X., Dai, Z., Zou, X. (2014). A Label-Free and PCR-Free Electrochemical Assay for Multiplexed MicroRNA Profiles by Ligase Chain Reaction Coupling with Quantum Dots Barcodes. Biosens. Bioelectron. 53, 414-419.

Table S3. Results for the determination of the let-7a miRNA in dilution of human serum. Related to Figure 5.

Samples	Spiked	Measure (n = 3)	Recovery (%)
1000-fold dilution human serum	50 nM	48.6 nM	97.2
	100 nM	96.7 nM	96.7
	250 nM	251.3 nM	100.5
10-fold dilution human serum	50 nM	50.6 nM	101.2
	100 nM	102.5 nM	102.5
	250 nM	248.7 nM	99.8

Data represented as mean ± SD.

Table S4. The detection of target and non-target miRNAs in serum sample via blind test. Related to Figure 5.

Samples	RAI[1] (%)	RSD (%) (n=3）	Output	Marked
1	100	8.2	let-7a miRNA	colon cancer patient1
2	92	6.7	let-7a miRNA	colon cancer patient2
3	96	3.5	let-7a miRNA	colon cancer patient3
4	28	4.3	Non-target	healthy person1
5	36	2.8	Non-target	healthy person2
6	47	4.6	Non-target	healthy person3

RAI is the relative absorption intensity that is calculated by the formula of $(A - A_0)/(A_t - A_0) \times 100\%$, where A, At, and A_0 represent the absorption intensity corresponding to non-target, target and Blank, respectively. RSD represents relative standard deviation. Data represented as mean ± SD.

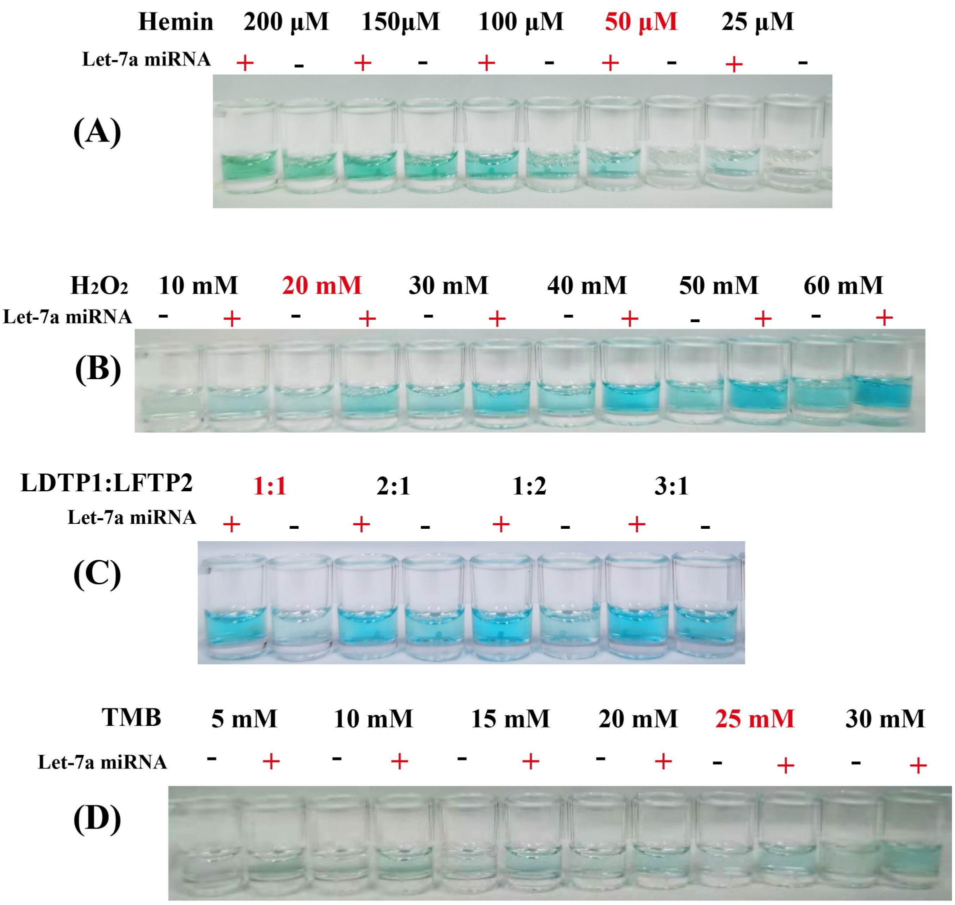

Figure S1. Photograph for color change with different amount of hemin, H_2O_2,the ratio of LDTP1 and LDTP2, and amount of TMB respectively. Related to Figure 2.

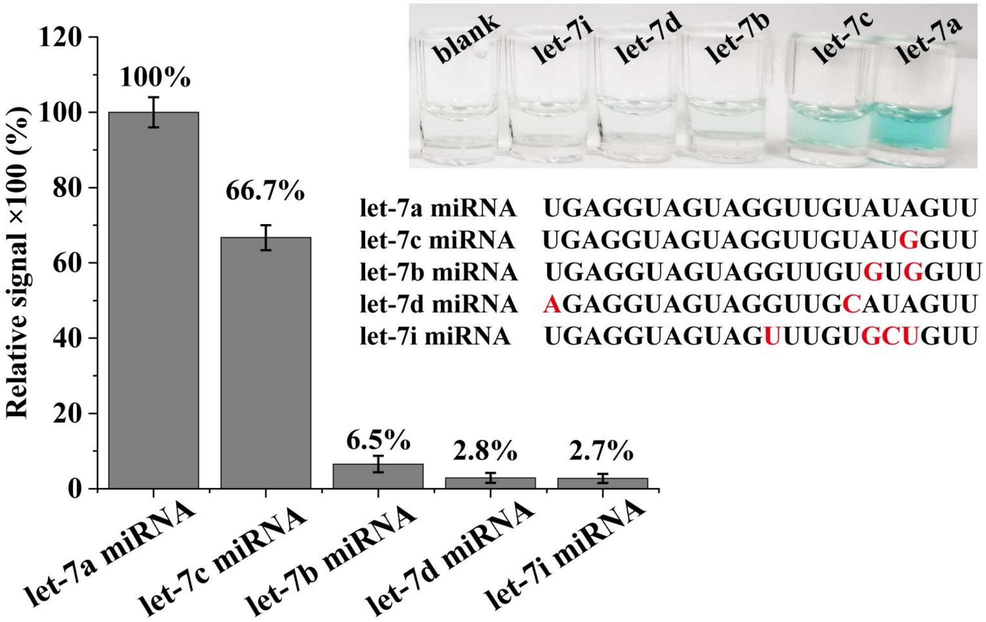

Figure S2. Specificity of the S-SDA for detection of different interferences, including homologous family RNA (let-7b, let-7c, let-7d or let-7i miRNA).The insets show the corresponding photograph of the color change. Data represented as mean ± SD. Related to Figure 3.
